# Maternal Choline Supplementation during Normal Murine Pregnancy Alters the Placental Epigenome: Results of an Exploratory Study

**DOI:** 10.3390/nu10040417

**Published:** 2018-03-28

**Authors:** Sze Ting (Cecilia) Kwan, Julia H. King, Jennifer K. Grenier, Jian Yan, Xinyin Jiang, Mark S. Roberson, Marie A. Caudill

**Affiliations:** 1 Division of Nutritional Sciences, Cornell University, Ithaca, NY 14850, USA; sk2563@cornell.edu (S.T.C.K.); jhk288@cornell.edu (J.H.K.); jy435@cornell.edu (J.Y.); xinyinjiang@brooklyn.cuny.edu (X.J.); 2 RNA Sequencing Core, Department of Biomedical Sciences, Cornell University, Ithaca, NY 14853, USA; jkg47@cornell.edu; 3 Department of Health and Nutrition Sciences, Brooklyn College, Brooklyn, NY 11210, USA; 4 Department of Biomedical Sciences, Cornell University, Ithaca, NY 14853, USA

**Keywords:** choline, placenta, imprinted genes, DNA methylation, microRNA

## Abstract

The placental epigenome regulates processes that affect placental and fetal development, and could be mediating some of the reported effects of maternal choline supplementation (MCS) on placental vascular development and nutrient delivery. As an extension of work previously conducted in pregnant mice, the current study sought to explore the effects of MCS on various epigenetic markers in the placenta. RNA and DNA were extracted from placentas collected on embryonic day 15.5 from pregnant mice fed a 1X or 4X choline diet, and were subjected to genome-wide sequencing procedures or mass-spectrometry-based assays to examine placental imprinted gene expression, DNA methylation patterns, and microRNA (miRNA) abundance. MCS yielded a higher (fold change = 1.63–2.25) expression of four imprinted genes (*Ampd3*, *Tfpi2*, *Gatm* and *Aqp1*) in the female placentas and a lower (fold change = 0.46–0.62) expression of three imprinted genes (*Dcn*, *Qpct* and *Tnfrsf23*) in the male placentas (false discovery rate (FDR) ≤ 0.05 for both sexes). Methylation in the promoter regions of these genes and global placental DNA methylation were also affected (*p* ≤ 0.05). Additionally, a lower (fold change = 0.3; *P_unadjusted_* = 2.05 × 10^−4^; FDR = 0.13) abundance of miR-2137 and a higher (fold change = 1.25–3.92; *p* < 0.05) expression of its target genes were detected in the 4X choline placentas. These data demonstrate that the placental epigenome is responsive to maternal choline intake during murine pregnancy and likely mediates some of the previously described choline-induced effects on placental and fetal outcomes.

## 1. Introduction

Epidemiological studies have shown that an adverse in utero environment is associated with a higher risk of developing obesity, metabolic syndrome, cardiovascular disease and other chronic diseases later in life [1,2,3]. These findings have led to the fetal programming hypothesis, which suggests that the developing fetus will generate an adaptive response to the suboptimal prenatal environment in order to increase its survival. This adaptive response involves changing the development of the placenta [4] as well as the fetus and its organs, with lasting effects on their functioning [3]. Although there is a growing body of evidence in support of this hypothesis, the molecular mechanisms mediating the programming phenomenon are less clear. One proposed mechanism linking prenatal exposure to later health outcomes is the modulation of gene expression via epigenetic processes.

Many epigenetic processes are involved in the regulation of gene expression. The most widely studied process is DNA methylation, the addition of methyl groups to the cytosine residues of cytosine-phosphate-guanine (CpG) dinucleotides situated within the regulatory regions of some genes (e.g., the promoter and the differential methylation region). One group of genes known to be regulated by DNA methylation is the imprinted genes, which are expressed according to parental origin [5,6]. Many of the imprinted genes are expressed in the placenta where they synthesize proteins that control the cell cycle, cell signaling and vascularization, and the uptake, utilization and storage of nutrients [5,6]; all of which can affect placental nutrient supply efficiency. Aberrant expression of these imprinted genes, including *IGF2*, *H19* and *TFPI2*, in the human placenta alters fetal growth and affects birth weight [5,7]. The expression of placental imprinted genes may also serve as a biomarker for future health outcomes, such as infant neurodevelopment and bone health at the age of four [8,9].

Another epigenetic mechanism that has received increased attention in recent years involves the microRNAs (miRNAs). These are small non-coding RNA molecules with 19–24 nucleotides that post-transcriptionally regulate gene expression [10]. Specifically, miRNA base-pairs with its mRNA targets in a sequence-specific manner to trigger mRNA transcript degradation or translational repression [10,11]. As such, expression of a miRNA will reduce the expression of its mRNA targets. The placenta produces many miRNAs, and their target genes are involved in cell proliferation, apoptosis, invasion and angiogenesis [11,12], which are essential to normal placental morphological and vascular development. Some miRNAs also regulate immune cell development at the maternal–fetal interface and mediate the immune response and maternal tolerance to the fetus [12]. Given their roles in many aspects of placental development and function, it is not surprising that miRNA dysregulation is associated with pregnancy disorders that impair fetal growth [10,13,14,15].

Choline is an essential micronutrient involved in one-carbon metabolism. Its oxidative derivative, betaine, converts homocysteine to methionine, which is used to generate *S*-adenosylmethionine (SAM), the universal methyl donor for many cellular methylation reactions including DNA methylation, which can exert lasting effects on gene expression and physiological processes [16]. Our previous studies indicated that maternal 4X choline supplementation during normal murine pregnancy improves placental vascularization and perfusion [17,18] and enhances placental nutrient supply [19] and early fetal growth [20]. Given the roles of imprinted genes, DNA methylation, and miRNAs in these placental and fetal outcomes (as described above), we sought to test the hypothesis that maternal choline supplementation would alter the placental epigenome during normal murine pregnancy. We tested this hypothesis using placental tissues that had been previously collected at embryonic day (E) 15.5—a time when the placenta reaches its maximal size and the fetus is rapidly growing [21,22]—from dams receiving 1X or 4X choline supplementation.

## 2. Materials and Methods

### 2.1. Animals and Diets

This study was an extension of an animal feeding study where we examined the impact of maternal choline supplementation on placental vascularization and nutrient transport system [17,19]. Briefly, adult non-Swiss Albino mice (Harlan) were purchased and used as a breeding colony. The mice had *ad libitum* access to commercial rodent chow and water, and were housed in microisolator cages (Ancare) in an environmentally-controlled room (22–25 °C, 70% humidity) with a 12-h light–dark cycle. Offspring from the breeding colony were fed a 1X choline diet (1.4 g choline chloride/kg diet; Dyets #103345) subsequent to weaning at 3 weeks old. Five days prior to mating, female mice were randomized to one of three diets: 1X choline, 2X choline (2.8 g choline chloride/kg diet; Dyets #103346), or 4X choline (5.6 g choline chloride/kg diet; Dyets #103347). Presence of a vaginal plug was defined as E0.5. Pregnant female mice continued their diet until they were euthanized using carbon dioxide at one of four time points: E10.5, E12.5, E15.5 or E18.5. The present study used the tissues collected at E15.5 from dams fed the 1X choline and 4X choline diets (*n* = 3 dams per group, per fetal sex; total *n* = 12 dams). To ensure statistical independence, only one placenta (either male or female) from each dam was used for each experiment. Each placenta, therefore, was considered to be an experimental unit. The 4X choline was chosen because our previous findings indicated a pronounced effect of this dosage on placental development and function [17,19], while E15.5 was chosen because it represents the time when the placenta reaches its maximal size and the fetus is rapidly growing [21,22]. To minimize maternal decidual contamination, the maternal decidua was carefully trimmed and removed during the dissection. The placental disks that were used for the experiments were washed multiple times with phosphate-buffered saline (PBS) to ensure that maternal blood was completely removed before they were weighed, flash frozen in liquid nitrogen and stored at −80 °C. The fetuses were also collected and weighed during the dissection and were flash frozen in liquid nitrogen before storage at −80 °C. The data on fetal weight, placental weight and placental efficiency were reported in [19]. All animal protocols were approved by the Institutional Animal Care and Use Committees at Cornell University (the approval number is 2001-0034) and were conducted in accordance with the Guide for the Care and Use of Laboratory Animals.

### 2.2. Fetal Sex Genotyping

Fetal DNA was extracted using the DNeasy blood and tissue kit (Qiagen, Germantown, MD, USA, Catalog #: 69506), and fetal sex was determined by PCR for the *Sry* gene, using forward 5′-TGGGACTGGTGACAATTGTC-3′ and reverse 5′-GAGTACAGGTGTGCAGCTCT-3′ primers. 

### 2.3. Placental RNA Extraction

Total RNA was extracted from the placentas using Trizol (Thermo Fisher, Waltham, MA, USA, Catalog #: 15596026) according to the manufacturer’s instructions with the following modifications: (i) an extra chloroform extraction step of the aqueous layer after the first phase separation; (ii) addition of 1 µL Glyco-blue (Thermo Fisher, Waltham, MA, USA, Catalog #: AM9515) before the isopropanol precipitation; and (iii) two washes of the RNA pellet with 75% ethanol. RNA concentration and purity were determined using a NanoDrop spectrophotometer (Thermo Fisher, Waltham, MA, USA). RNA integrity and presence of small RNAs (<<200 nucleotides) were determined with a Fragment Analyzer (Advanced Analytical, Ankeny, IA, USA).

### 2.4. Placental mRNA Sequencing and Data Analysis

The following procedures were conducted by the Cornell RNA Sequencing Core (Ithaca, NY, USA). NEBNext Ultra Directional RNA Library Prep Kit (New England Biolabs, Ipswich, MA, USA, Catalog #: E7420) was used to make polyA+ RNAseq libraries, using 1 µg total RNA input. RNAseq libraries were sequenced on an Illumina NextSeq500 with single-end 81 nt reads to generate an average of 44 M (min 37 M) reads per sample. Raw reads were trimmed and filtered with cutadapt [23] (parameters: -m 20 -q 20 -a AGATCGGAAGAGCACACGTCTGAACTCCAGTC --match-read-wildcards) to remove adaptor and low quality bases. After processing, reads were mapped to the reference mouse transcriptome (UCSC mm10) with Tophat (Version 2.0) [24] (parameters: -G <UCSC_mm10.gtf> --no-novel-juncs --library-type fr-firststrand). FPKM (fragments per kilobase of transcript per million mapped reads) values were generated, and statistical analyses (both females and males together as well as separately) were performed using Cuffdiff (Cufflinks, Version 2.2) [25] (parameters: --library-type fr-firststrand). Genes were considered to have significantly different expression when the false discovery rate (FDR) was less than 0.2. Differentially-expressed imprinted genes were identified as listed on the MRC Harwell Imprinting Webpages (http://www.har.mrc.ac.uk/research/genomic_imprinting/) [26]. RNAseq data have been deposited in the National Center for Biotechnology Information (NCBI)’s Gene Expression Omnibus (GEO) and are accessible through the GEO Series accession number, GSE111296 (https://www.ncbi.nlm.nih.gov/geo/query/acc.cgi?acc=GSE111296).

### 2.5. Placental miRNA Sequencing and Data Analysis

The following procedures were conducted by the Cornell RNA Sequencing Core (Ithaca, NY, USA). Libraries were made using the NEBNext Small RNA Library Prep Kit (New England Biolabs, Ipswich, MA, USA, Catalog #: E7330) using 1 µg total RNA input and were size-selected for 18–30 nt inserts on polyacrylamide gels. Small RNA libraries were sequenced on an Illumina HiSeq2500 with single-end 50 nt reads to generate an average of 12 M (min 7.5 M) reads per sample. Raw reads were trimmed and filtered with cutadapt [23] (parameters: -m 10 -q 20 -a AGATCGGAAGAGCACACGTCTGAACTCCAGTC --match-read-wildcards) to remove adaptor and low quality bases. Processed reads were mapped to mature mouse miRNAs (miRBase, Version 21) using miRDeep2 [27]. One female placental sample in the 1X choline group had a lower miRNA-mapped read frequency, suggesting possible sample degradation. Therefore, this sample was excluded from subsequent analyses. Small RNA sequencing data have been deposited in NCBI’s Gene Expression Omnibus and are accessible through GEO Series accession number GSE111296 (https://www.ncbi.nlm.nih.gov/geo/query/acc.cgi?acc=GSE111296).

Statistical testing of differential expression of the miRNAs was determined in EdgeR using a generalized linear model with a negative binomial distribution and filtering of CPM (counts per million) > 1 in at least 1 sample. Predicted mRNA targets of the most differentially-expressed miRNA were identified using TargetScan (Version 7.1) [28]. Strong targets were defined, in a similar manner to other investigations, with a context++ score ≤ −0.2, where a more negative score indicates greater repression [29,30]. Gene ontology was conducted for the mRNA targets using PANTHER Overrepresentation Test (Version 11.1) [31,32]. The expression of each of the mRNA targets identified from TargetScan was obtained from the mRNA sequencing dataset.

### 2.6. Placental DNA Extraction

Genomic DNA was extracted from the placenta using the DNeasy blood and tissue kit (Qiagen, Germantown, MD, USA, Catalog #: 69506), with the addition of RNase A (Qiagen, Germantown, MD, USA, Catalog #: 19101) to remove any co-purified RNA. DNA concentration and purity were evaluated using a NanoDrop spectrophotometer.

### 2.7. Promoter Methylation Assay and Data Analysis

A site-specific methylation analysis was conducted to determine the methylation patterns in the promoter regions of the differentially-expressed placental imprinted genes identified from the mRNA sequencing dataset. This assay was performed by the Cornell Epigenomics Core (New York, NY, USA) using the EpiTyper MassArray System (Agena Bioscience, San Diego, CA, USA), as described previously [33]. Briefly, genomic DNA was bisulfite-treated using the EZ DNA methylation kit (Zymo Research, Irvine, CA, USA, Catalog #: D5001). PCR amplification was performed to amplify the regions of interest using bisulfite-treated DNA and T7-promoter tagged primers (Supplemental Table S1), followed by Shrimp Alkaline Phosphatase (SAP) treatment and in vitro transcription. The resulting products were then loaded onto a SpectroCHIP Array and analyzed by a matrix-assisted laser desorption/ionization time-of-flight mass spectrometer (MALDI-TOF MS). Percent methylation of the cytosine residues at each CpG unit, which may contain more than one CpG site, was obtained from the EpiTyper software. CpG units in the promoter region of each gene were labelled alphabetically with letters. For quality control purposes, CpG units that could not be reliably detected by EpiTyper (e.g., a high signal to noise ratio, a high or low mass, duplication, inadequate number of replicates) were excluded from further analyses (Supplemental Table S2) [34]. The methylation level at each individual CpG unit as well as the average methylation of all the CpG units for each gene were analyzed using one-way ANOVA, where *p* ≤ 0.05 indicated statistical significance. Data are presented as means with 95% confidence intervals. All the statistical analyses were done using SPSS software (Version 23, IBM, Armonk, NY, USA).

### 2.8. Global DNA Methylation Assay and Data Analysis

Global DNA methylation was measured using LC-MS/MS (Thermo Fisher, Waltham, MA, USA), as described previously, with modifications based on our instrumentation [34,35]. Briefly, 300 ng genomic DNA was digested with nuclease P1 (Sigma-Aldrich, St. Louis, MO, USA, Catalog #: N8630), followed by digestion with phosphodiesterase 1 (Sigma-Aldrich, St. Louis, MO, USA, Catalog #: P3243) and digestion with alkaline phosphatase (Sigma-Aldrich, St. Louis, MO, USA, Catalog #: P4252). Samples were diluted with 0.1% formic acid in water and injected into the instrument for analysis. Global methylation is presented as a percentage of the amount of 5-methyl-2′-deoxycytidine (5mdC) relative to the total amount of cytosine (i.e.,: 5mdC/(dC + 5mdC)). Data were first analyzed without stratification by fetal sex and then separately for each fetal sex using one-way ANOVA. Statistical significance was defined as *p* ≤ 0.05. Data are presented as means with 95% confidence intervals. All the statistical analyses were done using SPSS software (Version 23, IBM, Armonk, NY, USA).

## 3. Results

### 3.1. Placental Imprinted Gene Expression

Supplementation during pregnancy with 4X maternal choline altered (FDR < 0.2) the placental expression of 131 genes, 28 of which were common to both male and female placentas. Because placental gene expression exhibits sexual dimorphism [36], we also analyzed the male and female placentas separately. In the female placentas, 4X (versus 1X) choline decreased the expression of 44 genes (FDR < 0.2) and increased the expression of 143 genes (FDR < 0.2). Among these 187 genes exhibiting differential expression, three were known imprinted genes: *Tfpi2* (fold change = 2.17), *Ampd3* (fold change = 1.63) and *Gatm* (fold change = 1.65) (Table 1). Furthermore, *Aqp1*, a recently suggested placenta-specific imprinted gene [37], was also affected by 4X choline supplementation (fold change = 2.25) (Table 1). In the male placentas, 4X (vs. 1X) choline decreased the expression of 79 genes (FDR < 0.2) and increased the expression of 62 genes (FDR < 0.2). Among these 141 genes affected by 4X choline, three were known to be imprinted genes: *Qpct* (fold change = 0.46), *Dcn* (fold change = 0.58) and *Tnfrsf23* (fold change = 0.62) (Table 2). It is worth noting that all these placental imprinted genes remained statistically significant with a lower FDR threshold (e.g., FDR < 0.1). Regression analyses indicated positive correlations of the imprinted genes expression in the male placentas with the weight of the male placentas (*Qpct*: β = 0.00079, *p* = 0.046; *Dcn*: β = 0.000049, *p* = 0.01; *Tnfrsf23*: β = 0.0003, *p* = 0.039). Non-significant associations were also detected between these imprinted genes and the efficiency of the male placentas (*Dcn*: β = −0.00061, *p* = 0.09; *Tnfrsf23*: β = −0.0041, *p* = 0.078).

### 3.2. Placental Gene Promoter Methylation

**Figure 1:**
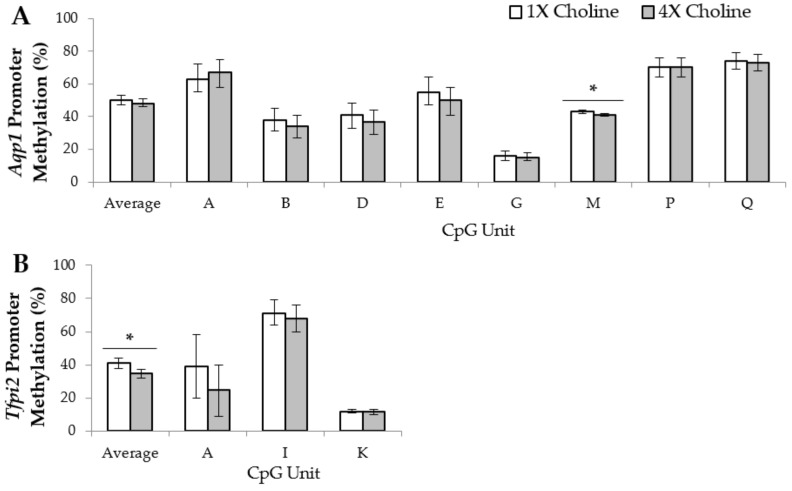

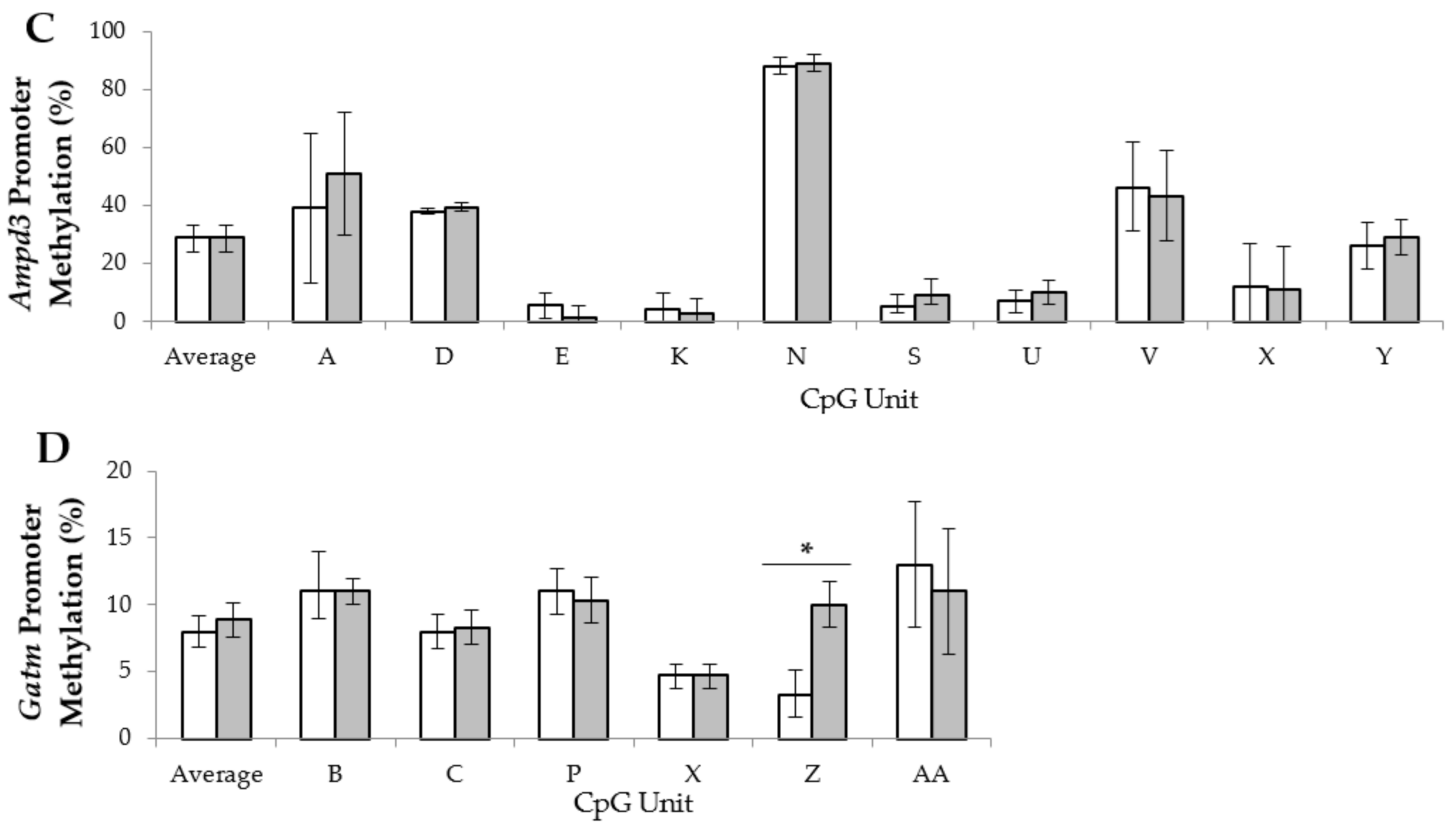
Average percentage of cytosine-phosphate-guanine (CpG) methylation across the promoter region and the percentage of methylation of each CpG unit within the promoter region of (**A**) *Aqp1*; (**B**) *Tfpi2*; (**C**) *Ampd3*; and (**D**) *Gatm* in the female placentas from dams in the 1X and 4X choline groups. Each CpG unit may contain more than one CpG site. Only CpG units with measurements that met the quality control criteria were included in the final analyses. One placenta (either male or female) from each dam (*n* = 3 dams per treatment, per fetal sex) was used, and each placenta was considered to be a statistical unit in the statistical analysis. Data were analyzed using one-way ANOVA and are presented as means with 95% confidence intervals. * *p* ≤ 0.05.

Because promoter DNA methylation is frequently involved in regulating gene transcription, promoter methylation patterns of the differentially-expressed imprinted genes were examined. In the female placentas, *Aqp1* had eight CpG units that met our quality control criteria and were analyzed. Compared to 1X choline, 4X choline supplementation yielded lower (*p* = 0.026) methylation of CpG unit M in the promoter region of *Aqp1*, but had no effects on the other CpG units. For *Tfpi2*, three CpG units in the promoter region met our quality control criteria and were included in the final analysis. Although the changes in the methylation of each individual CpG unit did not achieve statistical significance, the average CpG methylation in the promoter region was significantly lower (*p* = 0.009) in response to 4X choline supplementation (vs. 1X choline). For *Gatm*, among the six CpG units examined in this study, the methylation of CpG unit Z was three times higher (*p* = 0.009) in the female placentas of the 4X choline group than those of the 1X choline group. None of the other CpG units in the *Gatm* promoter were significantly affected by 4X choline supplementation, nor was methylation altered in the promoter region of *Ampd3* (*p* > 0.05) (Figure 1).

**Figure 2:**
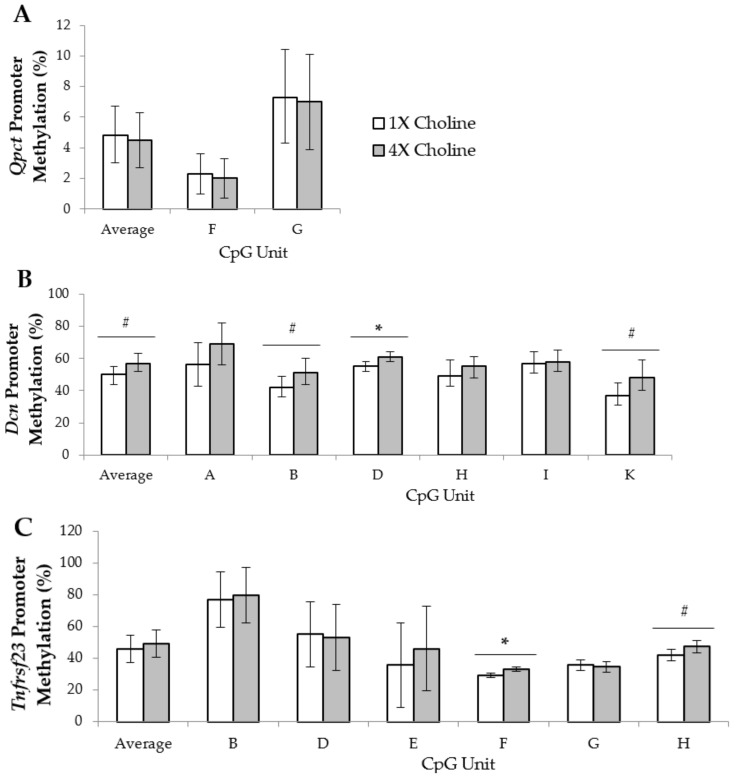
Average percentage of cytosine-phosphate-guanine (CpG) methylation across the promoter region and percentage of methylation of each CpG unit within the promoter regions of (**A**) *Qpct*; (**B**) *Dcn* and (**C**) *Tnfrsf23* in male placentas from dams in the 1X and 4X choline groups. Each CpG unit may contain more than one CpG site. Only CpG units with measurements that met the quality control criteria were included in the final analyses. One placenta (either male or female) from each dam (*n* = 3 dams per treatment, per fetal sex) was used, and each placenta was considered to be a statistical unit in the statistical analysis. Data were analyzed using one-way ANOVA and are presented as means with 95% confidence intervals. * *p* ≤ 0.05. # 0.05 < *p* < 0.1.

In the male placentas, six CpG units in the *Dcn* promoter met our quality control criteria and were included in the analysis. Supplementation with 4X choline yielded higher (*p* = 0.022) methylation of CpG unit D compared to 1X choline supplementation. In addition, trends for higher methylation of CpG units B (*p* = 0.068) and K (*p* = 0.063), as well as average methylation in the promoter region (*p* = 0.053) were detected in response to 4X choline supplementation. For *Tnfrsf23*, six CpG units in the promoter region were analyzed. Among these CpG units, methylation of CpG unit F was higher (*p* = 0.007) in the placentas of dams given 4X choline compared to those given 1X choline. Methylation of CpG unit H also trended higher (*p* = 0.052) in the 4X choline placentas vs 1X choline placentas. In contrast, the methylation in the *Qpct* promoter appeared unaffected (*p* > 0.05) by maternal choline supplementation (Figure 2).

### 3.3. Placental Global DNA Methylation

**Figure 3:**
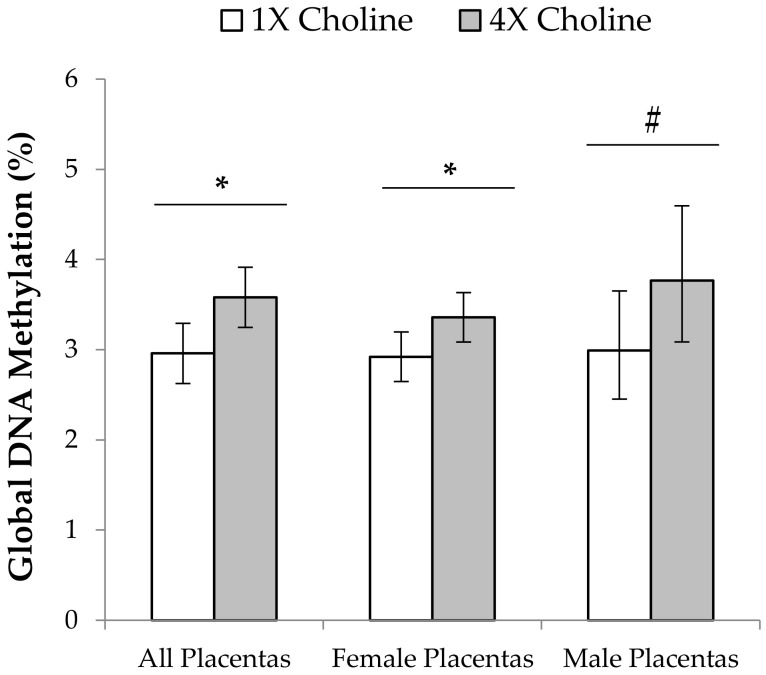
Percentage of global DNA methylation in the placentas from dams in the 1X and 4X choline groups. One placenta (either male or female) from each dam (*n* = 3 dams per treatment, per fetal sex) was used, and each placenta was considered to be a statistical unit in the statistical analysis. Data were first analyzed without stratification by fetal sex and then separately for each fetal sex using one-way ANOVA. Results are presented as means with 95% confidence intervals. * *p* ≤ 0.05. # *p* = 0.086.

Regardless of fetal sex, global placental DNA methylation was 21% higher (*p* = 0.015) in the 4X choline group compared to the 1X choline group. In the female placentas, 4X choline supplementation yielded higher (*p* = 0.035) global placental DNA methylation compared with 1X choline supplementation. Global DNA methylation in the female placentas also positively correlated with fetal weight and placental efficiency (fetal weight: β = 0.2, *p* =0.024; placental efficiency: β = 3.95, *p* = 0.037). A numerically higher abundance of global DNA methylation was also detected in the male placentas in response to 4X choline versus 1X choline (Figure 3); however, this numerical difference did not achieve statistical significance (*p* = 0.086). No significant associations (*p* ≥ 0.16) were found between global DNA methylation in male placentas and male fetal or placental outcomes.

### 3.4. Placental miRNA Expression

Following data processing, 609 miRNAs were included in the final statistical analyses. Among these miRNAs, the expression of miR-2137 was found to be significantly lower (fold change = 0.3; FDR = 0.125, *P*_unadjusted_ = 2.05 × 10^−4^) in response to 4X versus 1X maternal choline supplementation. Although significance was not achieved upon stratification by fetal sex, both female and male placentas from the 4X choline group exhibited lower expression of miR-2137 (fold change = 0.4 and 0.3, respectively). Based on TargetScan [28] prediction, miR-2137 has 170 mRNA targets with context++ score ≤−0.2. These mRNA targets are related to 11 biological processes (Table 3) based on GO term analysis with PANTHER [31,32]. To determine if any of these predicted mRNA targets were affected by placental reduction of miR-2137, their expression was assessed using the data generated from the mRNA-sequencing experiment. Because the predicted mRNA targets constitute only a small number of the genes listed on the mRNA sequencing dataset, mRNA targets with an unadjusted *p* value of ≤ 0.05 are reported. The expression of five of the predicted mRNA targets was higher in the female placentas supplemented with 4X choline compared to those supplemented with 1X choline (Table 4). These included *Cd109* (fold change = 1.55; *p* < 0.01), *Mt3* (fold change = 3.85; *p* < 0.01), *Plg* (fold change = 3.92; *p* < 0.01), *Gja4* (fold change = 1.33; *p* = 0.01), and *Psrc1* (fold change = 1.49; *p* = 0.05). In the male placentas, 4X choline supplementation resulted in higher expression of four of the predicted mRNA targets (Table 4), which were *Pmaip1* (fold change = 1.39; *p* = 0.02), *Pcdh1* (fold change = 1.25; *p* = 0.04), *Mt3* (fold change = 1.69; *p* = 0.04), and *Cd28* (fold change = 1.64; *p* = 0.05). In contrast to the female placentas, the expression of *Gja4* in the male placentas of 4X choline was downregulated (fold change = 0.58; *p* < 0.01).

## 4. Discussion

To the best of our knowledge, this is the first study to survey the effects of maternal choline supplementation on epigenetic markers in the mouse placenta in a genome-wide manner. We demonstrated choline-induced sex-specific effects on imprinted gene expression patterns, some of which are associated with changes in promoter region CpG dinucleotide methylation. We also showed higher levels of global DNA methylation and suppression of miR-2137 abundance in placentas from dams supplemented with additional choline during gestation.

### 4.1. Maternal Choline Supplementation Alters the Expression of Several Imprinted Genes in the Placenta

Placental expression of several imprinted genes was altered in a sex-specific manner in response to higher maternal choline intake during gestation. In the female placentas, maternal choline supplementation upregulated the expression levels of *Gatm*, *Tfpi2*, *Aqp1* and *Ampd3*. *Gatm* (glycine amidinotransferase) diverts glycine into the biosynthesis of creatine, which is a source of energy for tissues with high energy needs. Lower expression of *Gatm* in the brain is associated with mental and behavioral impairments whereas lower placental *Gatm* abundance is associated with intrauterine growth restriction [38,39]. As such, the choline-induced upregulation in placental *Gatm* abundance observed in the present study would be expected to improve fetal development. The other choline-altered imprinted genes in the female placentas are implicated in processes essential to normal placental vascular development and thereby also have the potential to affect fetal development. *Tfpi2* (tissue factor pathway inhibitor 2) inhibits the activity of matrix metalloproteinases and regulates placental perfusion [40,41], while deficiency of *Aqp1* (aquaporin 1) causes aberrant placental vascularization and fetal overgrowth [37]. Although the consequences of altered expression of *Ampd3* (AMP deaminase 3), a known regulator of fetal muscle and liver development [42], in the placenta remain to be examined, *Ampd3* deficiency in cancer cells has been shown to inhibit cell proliferation and invasion [43]. Collectively, the choline-induced upregulation of all these placental imprinted genes would be expected to improve placental vascularization and perfusion, likely contributing to the choline-induced beneficial effects on placental transport efficiency and fetal development reported previously [17].

The downregulation of the imprinted genes, *Dcn* (decorin) and *Tnfrsf23* (tumor necrosis factor receptor superfamily, member 23), in the male placentas in response to maternal choline supplementation may also result in an improved placental vascular network. *Dcn* encodes a small leucine-rich proteoglycan protein, and reduced placental *Dcn* expression enhances endothelial cell migration and remodeling of the placental vasculature [44]. *Tnfrsf23* is a receptor that binds to the cytotoxic tumor necrosis factor-related apoptosis-inducing ligand (TRAIL), and its downregulation may reduce apoptosis and modulate inflammatory responses during trophoblast invasion [45,46]. Additionally, in the male placentas, 4X choline downregulated the abundance of *Qpct* (glutaminyl cyclase), which controls placental nutrient delivery [47]. Upregulation of *Qpct* is frequently detected in pre-eclamptic placentas [48], possibly as a compensatory response to poor placental perfusion. Therefore, the choline-induced downregulation of *Qpct* in the present study may indicate sufficiently perfused placentas, which is consistent with the previously reported choline-induced enlargement of spiral arteries in the maternal decidua [17]. In sum, maternal choline supplementation altered the abundance of placental imprinted genes in a manner that is consistent with previous reports of choline-induced improvements in placental vascular development [17].

### 4.2. Altered Promoter-Region Methylation May Be One Epigenetic Mechanism Contributing to the Choline-Induced Differential Expression of the Placental Imprinted Genes

As expected, lower promoter methylation occurred concurrently with the upregulation of *Aqp1* and *Tfpi2* expression in the choline-supplemented female placentas, whereas higher methylation in the promoter regions occurred coincident with the downregulation of *Dcn* and *Tnfrsf23* in the choline-supplemented male placentas. However, higher methylation in the promoter of the upregulated *Gatm* was detected in the choline-supplemented female placentas. Although higher methylation generally results in transcriptional repression, exceptions have been noted in several genes where a methylated promoter still allows active gene transcription due to the presence of other regulatory factors that coordinate with promoter methylation to modulate transcription [49]. Additionally, because the imprinting of *Gatm* is associated with an unmethylated CpG island in its promoter region [50], the promoter hypermethylation observed in the present study may lead to a loss of imprinting and transcriptional activation of both of its maternal and paternal alleles, resulting in higher gene expression. Similarly, we observed that maternal choline supplementation altered the placental expression of *Qpct* and *Ampd3* without any noticeable changes in the methylation of their promoters. We suggest that the transcriptional regulation of these genes may be independent of promoter DNA methylation. A highly plausible explanation for the altered expression of these genes could involve modifications of the histone proteins. Indeed, others have reported that increased *Qpct* expression is associated with higher H3K4 methylation (e.g., H3K4me3) [47], whereas the expression of *Ampd3* is highly upregulated in mice depleted of *Hdac3* [51], one of the enzymes responsible for histone deacetylation that leads to a “closed” chromatin state and suppresses transcription. Notably, we have previously shown that doubling the maternal choline intake during the third trimester of human pregnancy alters global histone methylation and histone methyltransferase abundance in the placenta [34]. Taken together, the choline-induced alterations in the expression of the placental imprinted genes may be related to changes in the amount of methylation in different key transcriptional regulators.

### 4.3. Maternal Choline Supplementation Increases Global DNA Methylation in the Placenta

Similar to our previous findings in humans [34], higher global DNA methylation in both female and male placentas was found in response to additional maternal choline intake during pregnancy. Global DNA hypomethylation often leads to genomic instability that increases mutation frequency and disease susceptibility [52]. There is also a strong correlation between global DNA methylation in the placenta and fetal growth outcomes, where a 10-point increase in birth weight percentile is associated with 10% higher LINE-1 methylation (a surrogate marker of global DNA methylation) in the human placenta [53]. Our regression analyses also support a similar positive relationship between placental global DNA methylation with fetal body weight and placental efficiency in females. In sum, the choline-induced increase in placental global DNA methylation found in this study is expected to stabilize the placental genome, thereby minimizing any adverse effects on normal placental development and ultimately improving fetal development.

### 4.4. Maternal Choline Supplementation Reduces Placental miR-2137 Abundance, with Downstream Effects on the Expression of Its Potential Target Genes

Placental miR-2137 was downregulated by 4X choline supplementation in the present study. Based on the bioinformatics analyses, miR-2137 targets genes that are important for development, including the development of the mesodermal (GO: 0007498) and the ectodermal (GO: 0007398) layers. These primary germ layers give rise to different body organs, including muscles, bones, the cardiovascular system and the nervous system. Indeed, our bioinformatics analyses also indicated that miR-2137 impacts the development of these organs (GO: 0007399 and GO: 0007517). These findings not only support the proposed existence of a placental–brain axis and a placental–cardiovascular axis [54], but may also explain the effects of prenatal choline supply on programming offspring neurodevelopment [55] and normalizing the blood pressure of adult offspring from dams fed a low-protein diet throughout gestation [56]. In addition, these data provide a possible mechanism linking choline inadequacy to altered muscle membrane lipid composition and metabolism in mouse muscle cells [57] as well as disrupted bone homeostasis and a lower bone mass in mice and linear growth failure in young children [58,59]. Collectively, these data support the involvement of placental miR-2137 in mediating the effect of maternal choline intake on various fetal developmental outcomes.

Although miR-2137 has not been experimentally studied in the placenta, it has been examined in other tissues [60,61,62,63]. Consistent with our bioinformatics analyses, these studies showed that altered miR-2137 abundance changes the function of different organs, including the heart and the brain, as well as processes related to cell signaling and apoptosis (both of which lost statistical significance in our bioinformatics analyses upon adjustment for multiple testing). Interestingly, micronutrient supplementation in a paternal undernutrition mouse model also changes miR-2137 expression in the offspring pancreas [64], indicating that this miRNA may be particularly sensitive to nutritional status. In addition to the bioinformatics analyses, we found that choline-induced miR-2137 downregulation led to higher expression of several predicted mRNA targets. Despite being different between the female and male placentas, these genes all play a role in the vascular development (e.g., *Gja4* [65], *Mt3* [66] and *Plg* [67]), cell signaling (e.g., *Cd109* [68] and *Pcdh1* [69]) and apoptotic processes (e.g., *Psrc1* [70], *Pmaip1* [71,72] and *Cd28* [73]). These processes affect placental trophoblast survival and migration, and the remodeling of the maternal vasculature, ultimately affecting normal placental morphological and vascular development [74,75,76]. Taken together, changes induced by miR-2137 in response to a higher maternal choline intake may benefit placental development and offspring health.

### 4.5. Study Limitations

Our study had several limitations. First, only three samples per condition were used in this study; this was chosen based on tissue availability and conventional practices due to the uncertainty in sample size calculation when small fold changes are expected [77]. This may have limited our ability to detect additional placental imprinted genes and miRNAs that are developmentally important and responsive to maternal choline supplementation. Another potential limitation is the underestimation of the high variance in sequence reads distribution, which is likely due to the heterogeneity of the placental tissues as each cell type may have its own discrete gene expression profile that can confound the overall measurements. The issue of heterogeneity may also be relevant to the promoter methylation experiments [78]. Additionally, the number and magnitude of change in promoter methylation were relatively small, and their biological importance is currently unclear. It is possible that higher magnitude changes in the methylation of other epigenetic regulators (e.g., histones) or gene regions other than the promoter (e.g., the differential methylation region) are responsible for the choline-induced expression changes [49]. Measurements of SAM, the methyl donor directly involved in cellular methylation reactions, or choline metabolites in these placentas, may also provide additional insight into the changes observed in this study. However, these markers were not examined in the present study due to limited sample availability. With the exception of *Tfpi2* [79], the imprinted genes identified in the present study appear to be weakly imprinted in mouse placentas and may display biallelic expression in human placentas [80,81,82]; therefore, the relevance of these findings to human placentas is unknown. Although the pattern of differential gene expression appears to be sexually dependent, the reasons and mechanisms accounting for this observation remain to be elucidated. Because placental epigenetic markers are highly dependent on the developmental stage, it is also possible that different results may be found on different gestational days.

## 5. Conclusions and Future Directions

Findings from the present study add to the growing body of research that illustrates the responsiveness of the placental epigenome to maternal choline intake during pregnancy. Although there are limitations associated with the exploratory nature of this work, it is an important first step in elucidating the molecular mechanisms mediating some of the previously reported choline-induced effects on placental and fetal development. Additional studies are needed to address the functional effects of the placental epigenetic markers identified in this work, and to examine their clinical relevance in predicting pregnancy outcomes and offspring health.

## Figures and Tables

**Table 1:** Differentially-expressed imprinted genes in the female placentas in response to 4X versus 1X maternal choline supplementation ^1^.

Gene Symbol	Gene Name	Fold Change	False Discovery Rate (FDR) Value
*Aqp1* ^a^	aquaporin 1	2.25	0.009
*Tfpi2* ^b^	tissue factor pathway inhibitor 2	2.17	0.009
*Ampd3* ^b^	adenosine monophosphate deaminase 3	1.63	0.009
*Gatm* ^b^	glycine amidinotransferase	1.65	0.054

^1^ Data were derived from an RNA-sequencing dataset, and statistical analysis was performed using Cuffdiff (Cufflinks, Version 2.2) [25]. Genes with FDR < 0.2 were considered to have significantly different expression; ^a^ Imprinted gene identified by Guo et al. [37]; ^b^ Imprinted genes listed on the MRC Harwell Imprinting Webpages [26].

**Table 2:** Differentially-expressed imprinted genes in the male placentas in response to 4X versus 1X maternal choline supplementation ^1^.

Gene Symbol	Gene Name	Fold Change	FDR Value
*Qpct*	glutaminyl cyclase	0.46	0.012
*Dcn*	decorin	0.58	0.012
*Tnfrsf23*	tumor necrosis factor receptor superfamily, member 23	0.62	0.012

^1^ Data were derived from an RNA-sequencing dataset and statistical analysis was performed using Cuffdiff (Cufflinks, Version 2.2) [25]. Genes with FDR < 0.2 were considered to have significantly different expression. All the imprinted genes were identified as listed on the MRC Harwell Imprinting Webpages [26].

**Table 3:** Biological processes affected by the predicted mRNA targets of miR-2137 ^1^.

Biological Processes	*p*-Values
regulation of transcription from RNA polymerase II promoter (GO: 0006357)	<0.01
transcription from RNA polymerase II promoter (GO: 0006366)	<0.01
developmental process (GO: 0032502)	<0.01
muscle organ development (GO: 0007517)	0.01
transcription, DNA-dependent (GO: 0006351)	0.01
segment specification (GO: 0007379)	0.01
nervous system development (GO: 0007399)	0.01
system development (GO: 0048731)	0.02
RNA metabolic process (GO: 0016070)	0.02
mesoderm development (GO: 0007498)	0.05
ectoderm development (GO: 0007398)	0.05

^1^ Predicted mRNA targets, which have a context++ score ≤−0.2, were obtained from TargetScan (Version 7.1) [28] and were included in the gene ontology (GO) analysis. The gene ontology analysis was done using PANTHER (Version 11.1) [31,32] with Bonferroni correction, and *p* ≤ 0.05 was considered statistically significant.

**Table 4:** Predicted mRNA targets of miR-2137 that displayed upregulation in the female and male placentas in response to 4X choline supplementation ^1−3^.

**Female Placentas**	**Gene Symbol**	**Gene Name**	**Fold Difference**
	*Gja4*	gap junction protein, alpha 4	1.33
	*Psrc1*	proline/serine-rich coiled-coil 1	1.49
	*Cd109*	CD109 antigen	1.55
	*Mt3*	metallothionein 3	3.85
	*Plg*	plasminogen	3.92
**Male Placentas**	*Pcdh1*	protocadherin 1	1.25
	*Pmaip1*	phorbol-12-myristate-13-acetate-induced protein 1	1.39
	*Cd28*	CD28 antigen	1.64
	*Mt3*	metallothionein 3	1.69

^1^ Predicted mRNA targets, which have a context++ score ≤−0.2, were obtained from TargetScan (Version 7.1) [28]; ^2^ Fold change data were derived from an RNA-sequencing dataset, and statistical analysis was performed using Cuffdiff (Cufflinks, Version 2.2) [25]; ^3^ *P_unadjusted_* ≤ 0.05 was considered to be significant.

## Supplementary Materials

The following are available online at http://www.mdpi.com/2072-6643/10/4/417/s1, Table S1: Primers for promoter methylation assays and their targeted chromosomal region; Table S2: Selection of CpG units for each imprinted genes for statistical analyses.
