# Supplementary material for: Maternal Choline Supplementation during Normal Murine Pregnancy Alters the Placental Epigenome: Results of an Exploratory Study
Source: Nutrients. 2018 Mar 28;10(4):417. doi: 10.3390/nu10040417 (PMC5946202; doi:10.3390/nu10040417)
Supplement: Supplementary file 1 [file nutrients-10-00417-s001.pdf]

**Kwan et al.**
**Supplementary Materials**

**Table S1.** Primers for promoter methylation assays and their targeted chromosomal region[1]

| Gene | Primers and Target Sequence |
|---|---|
| *Aqp1* | **Primers:**<br>F: 5′-AGGAAGAGAGTTGTTTGGTTAAGGTTTGTTATTGT-3′<br>R: 5′-CAGTAATACGACTCACTATAGGGAGAAGGCTAAAACCAAAAAAATCCAAATATCC-3′<br>**Amplicon size:** 372 bp **Targeted chromosomal region:** chr6:55335767-55336139 **Total number of CpG sites:** 4 |
| | **Primers:**<br>F: 5′-AGGAAGAGAGGGTTTTTTGAGAAGAGTTAGTTTGG-3′<br>R: 5′-CAGTAATACGACTCACTATAGGGAGAAGGCTAAAATCATAACCAAAAACTCAACCA-3′<br>**Amplicon size:** 425 bp **Targeted chromosomal region:** chr6:55336133-55336558 **Total number of CpG sites:** 10 |
| *Tfpi2* | **Primers:**<br>F: 5′-AGGAAGAGAGGTTAGGTTTGATGTGAATGGTTTTT-3′<br>R: 5′-CAGTAATACGACTCACTATAGGGAGAAGGCTAAATATTTCACCAAATCATATTCAA-3′<br>**Amplicon size:** 493 bp **Targeted chromosomal region:** chr6:3970420-3970913 ('-' strand) **Total number of CpG sites:** 6 |
| | **Primers:**<br>F: 5′-AGGAAGAGAGTTTTTTTTGAATATGATTTGGTGAA-3′<br>R: 5′-CAGTAATACGACTCACTATAGGGAGAAGGCTAAACCCTAAAAACTTAACCACCAAC-3′<br>**Amplicon size:** 341 bp **Targeted chromosomal region:** chr6:3970109-3970450 ('-' strand) **Total number of CpG sites:** 5 |
| | **Primers:**<br>F: 5′-AGGAAGAGAGTTTGATTTTAGAGTTTATTGGGATGA-3′<br>R: 5′-CAGTAATACGACTCACTATAGGGAGAAGGCTTAAAAAAACAACCTTTAAACCACCC-3′<br>**Amplicon size:** 391 bp **Targeted chromosomal region:** chr6:3968386-3968777 ('-' strand) **Total number of CpG sites:** 13 |

| | |
|---|---|
| *Ampd3* | **Primers:**<br>F: 5′-AGGAAGAGAGGGTGTTTAGGTTTTTTGTTGGTA-3′<br>R: 5′-CAGTAATACGACTCACTATAGGGAGAAGGCTACCAAAAATACTACAAACTCACCA-3′<br>**Amplicon size:** 487 bp **Targeted chromosomal region:** chr7:110768615-110769102 **Total number of CpG sites:** 48 |
| *Gatm* | **Primers:**<br>F: 5′-AGGAAGAGAGGTTAAGGAGTAAGGGAGGGTTTGTA-3′<br>R: 5′-CAGTAATACGACTCACTATAGGGAGAAGGCTACAACCAATAAACCAAACCCTAAAC-3′<br>**Amplicon size:** 495 bp **Targeted chromosomal region:** chr2:122610841-122611336 **Total number of CpG sites:** 35 |
| | **Primers:**<br>F: 5′-AGGAAGAGAGTTAGGGTTTGGTTTATTGGTTGTTA-3′<br>R: 5′-CAGTAATACGACTCACTATAGGGAGAAGGCTTCAAACACCCAAATCATATTAAAAAA-3′<br>**Amplicon size:** 483 bp **Targeted chromosomal region:** chr2:122611314-122611797 **Total number of CpG sites:** 13 |
| *Qpct* | **Primers:**<br>F: 5′-AGGAAGAGAGTTTGTTTTTGATAGTTGGGAATTTG-3′<br>R: 5′-CAGTAATACGACTCACTATAGGGAGAAGGCTTAAACATCCATAAAAACACCCCTAA-3′<br>**Amplicon size:** 357 bp **Targeted chromosomal region:** chr17:79052008-79052365 **Total number of CpG sites:** 19 |
| *Dcn* | **Primers:**<br>F: 5′-AGGAAGAGAGGTAATTGTATTTGTTGGTGGGTTG-3′<br>R: 5′-CAGTAATACGACTCACTATAGGGAGAAGGCTACAAAAACATAAAATCATCACACAAT-3′<br>**Amplicon size:** 499 bp **Targeted chromosomal region:** chr10:97476606-97477105 **Total number of CpG sites:** 9 |

| | |
|---|---|
| *Tnfrsf23* | **Primers:**<br>F: 5′-AGGAAGAGAGGAGATGGTTGGAGAGATGATATAGTT-3′<br>R: 5′-CAGTAATACGACTCACTATAGGGAGAAGGCTCCTCACCCTTAAAAAACAAAAAAAA-3′<br>**Amplicon size:** 496 bp **Targeted chromosomal region:** chr7:143687074-143687570 ('-' strand) **Total number of CpG sites:** 8<br><br>**Primers:**<br>F: 5′-AGGAAGAGAGTAGGTGAGGGTAGATGGGTATAGGT-3′<br>R: 5′-CAGTAATACGACTCACTATAGGGAGAAGGCTAAAAACCTCAAAATATCCAAAACTC-3′<br>**Amplicon size:** 483 bp **Targeted chromosomal region:** chr7:143686439-143686922 ('-' strand) **Total number of CpG sites:** 4 |

[1] Primers were designed using EpiDesigner (http://www.epidesigner.com/start3.html). Information regarding amplicon size and the number of CpG sites was obtained from the output generated by EpiDesigner. Information regarding the chromosomal location was obtained from UCSC Genome Browser (Version mm10 for mouse).

**Table S2.** Selection of CpG units for each imprinted genes for statistical analyses

| Gene symbol | CpG units included | CpG units excluded[a] |
|---|---|---|
| *Aqp1* | A, B, D, E, G, M, P, Q | C, F, H, I, K, L (SN); J (H_mass); N, O (D) |
| *Tfpi2* | A, I, K | B, C, L, P (D); D-H, N (SN); J, Q (H_mass); M, O (IS) |
| *Ampd3* | A, D, E, K, N, S, U, V, X, Y | B, G, H, J, M (SN); C, F, L, P, R (IS); I, O, W, Z (H_mass); Q, T (D) |
| *Gatm* | B, C, P, X, Z, AA | A, D, E, G, I, J, O, R, T, AB, AC (SN); F, K (IS); H, L-N, Q, U, W (D); S, V, Y (H_mass) |
| *Qpct* | F, G | A, E (H_mass); B-D, J (D); H, K (SN); I (IS) |
| *Dcn* | A, B, D, H, I, K | C, E, G, J, L (SN); F (L_mass) |
| *Tnfrsf23* | B, D-H | A, C (SN); I (L_mass) |

[a] Reasons for exclusion are indicated in parentheses. SN, high signal to noise ratio; H_mass, the CpG unit had high mass and was not reliably detected; L_mass, the CpG unit had low mass and was not reliably detected; D, duplicate CpG unit; IS, inadequate number of successful replicate measurements.
